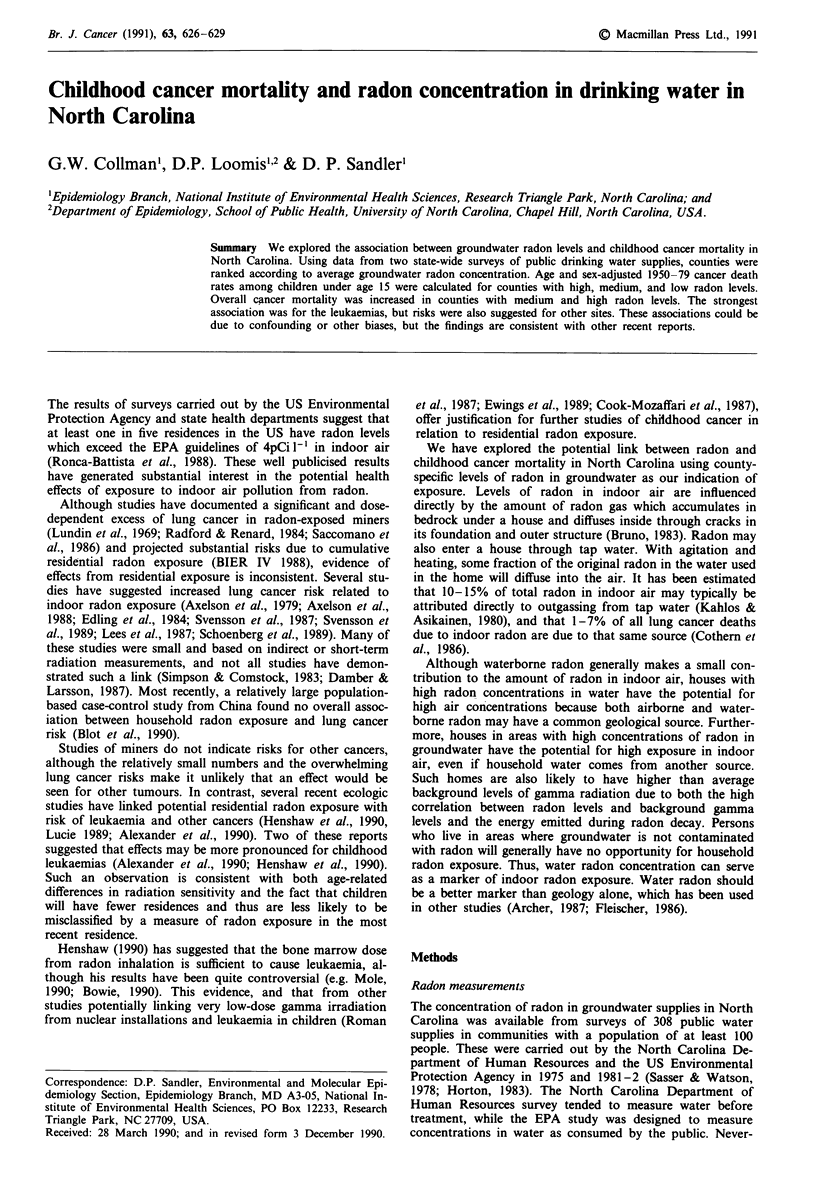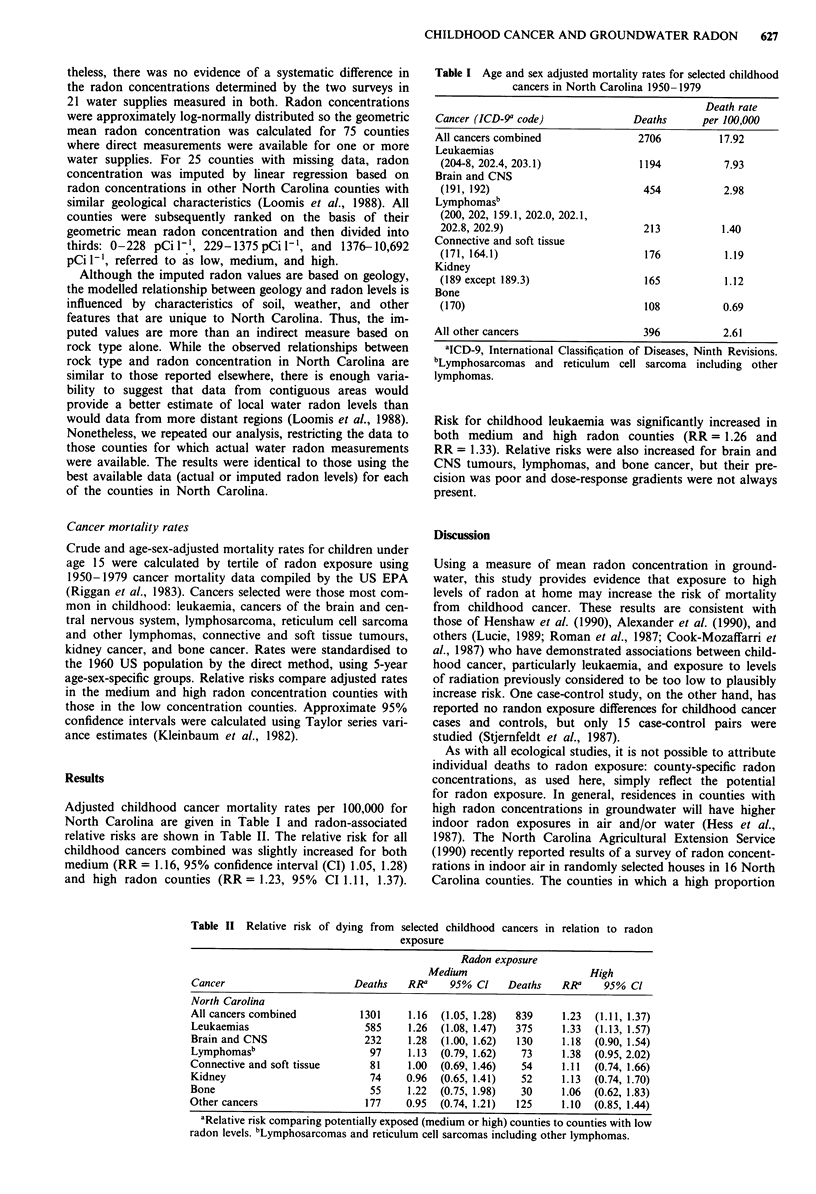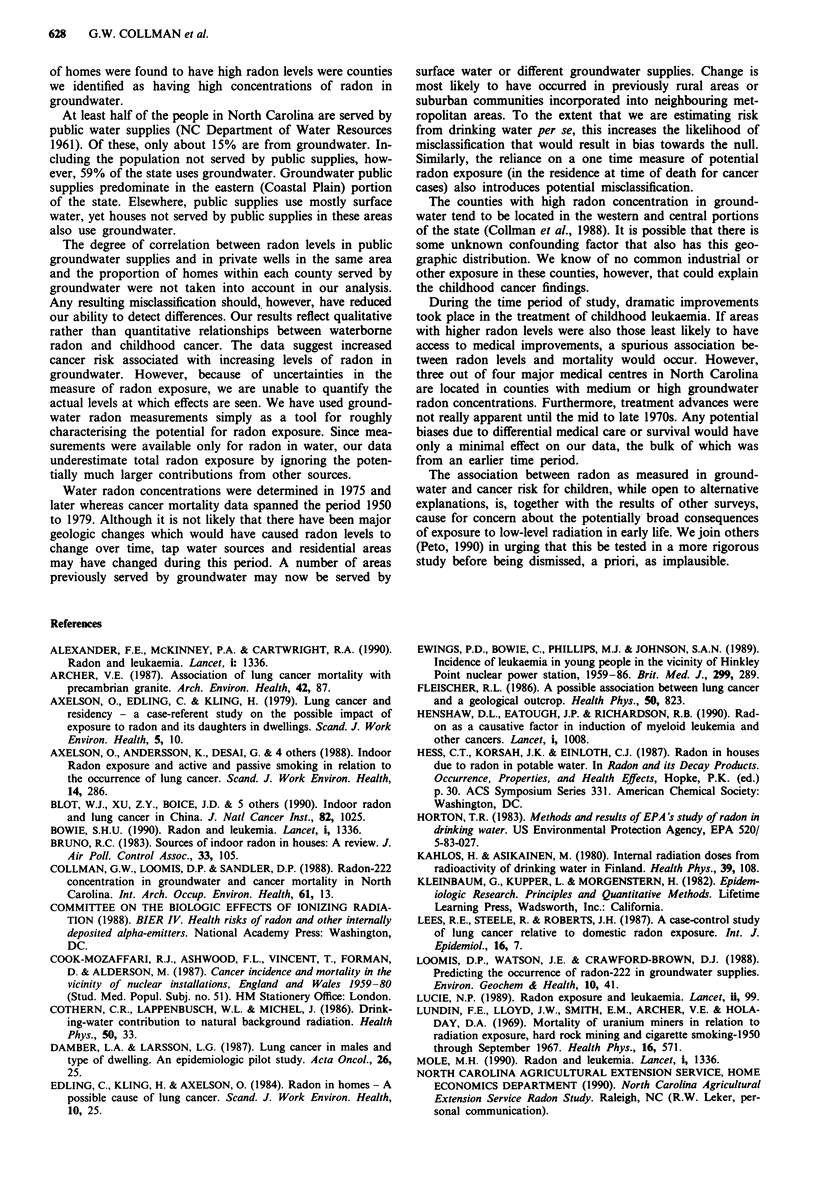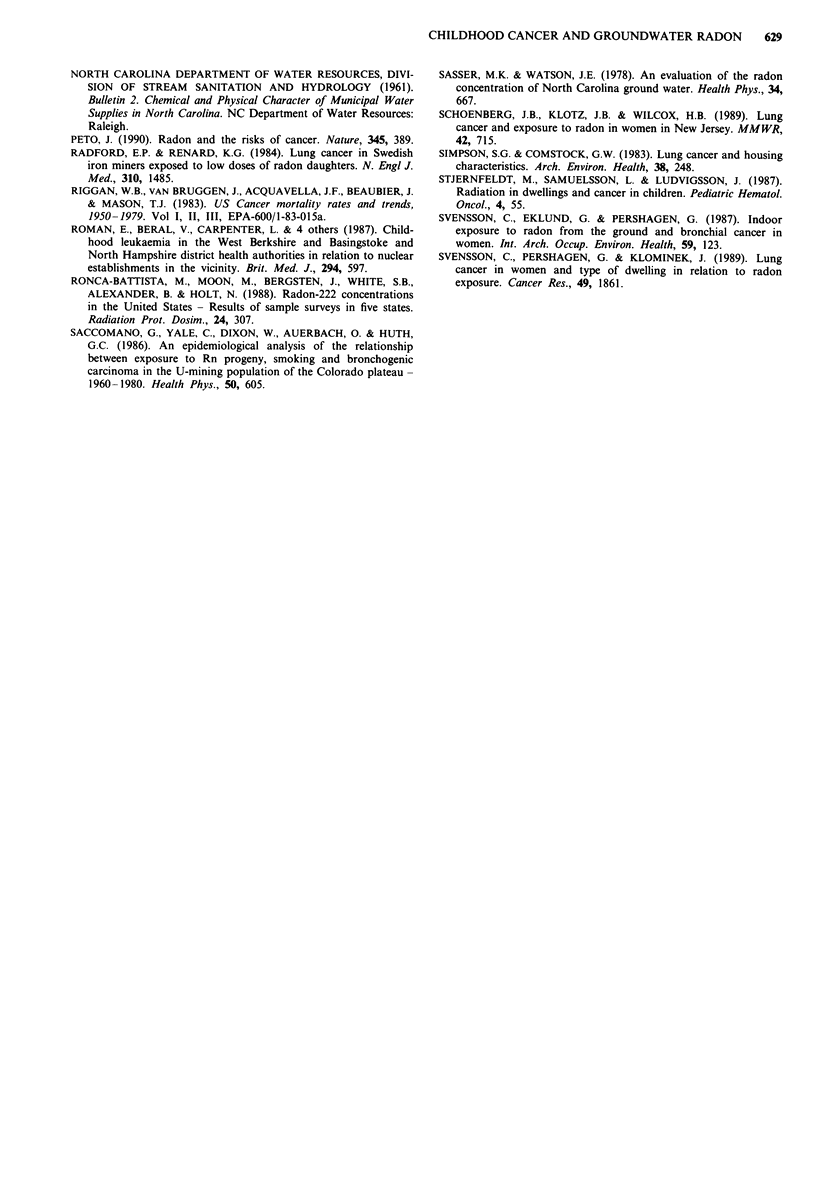# Childhood cancer mortality and radon concentration in drinking water in North Carolina.

**DOI:** 10.1038/bjc.1991.143

**Published:** 1991-04

**Authors:** G. W. Collman, D. P. Loomis, D. P. Sandler

**Affiliations:** Epidemiology Branch, National Institute of Environmental Health Sciences, Research Triangle Park, North Carolina 27709.

## Abstract

We explored the association between groundwater radon levels and childhood cancer mortality in North Carolina. Using data from two state-wide surveys of public drinking water supplies, counties were ranked according to average groundwater radon concentration. Age and sex-adjusted 1950-79 cancer death rates among children under age 15 were calculated for counties with high, medium, and low radon levels. Overall cancer mortality was increased in counties with medium and high radon levels. The strongest association was for the leukaemias, but risks were also suggested for other sites. These associations could be due to confounding or other biases, but the findings are consistent with other recent reports.


Br. J. Cancer (1991), 63, 626-629 © Macmillan Press Ltd., 1991

### Childhood cancer mortality and radon concentration in drinking water in North Carolina

G.W. Collman[1], D.P. Loomis[1,2] & D. P. Sandler[1]

[1]*Epidemiology Branch, National Institute of Environmental Health Sciences, Research Triangle Park, North Carolina; and* [2]*Department of Epidemiology, School of Public Health, University of North Carolina, Chapel Hill, North Carolina, USA.*

**Summary** We explored the association between groundwater radon levels and childhood cancer mortality in North Carolina. Using data from two state-wide surveys of public drinking water supplies, counties were ranked according to average groundwater radon concentration. Age and sex-adjusted 1950-79 cancer death rates among children under age 15 were calculated for counties with high, medium, and low radon levels. Overall cancer mortality was increased in counties with medium and high radon levels. The strongest association was for the leukaemias, but risks were also suggested for other sites. These associations could be due to confounding or other biases, but the findings are consistent with other recent reports.

The results of surveys carried out by the US Environmental Protection Agency and state health departments suggest that at least one in five residences in the US have radon levels which exceed the EPA guidelines of $4 pCi\,l^{-1}$ in indoor air (Ronca-Battista *et al.*, 1988). These well publicised results have generated substantial interest in the potential health effects of exposure to indoor air pollution from radon.

Although studies have documented a significant and dose-dependent excess of lung cancer in radon-exposed miners (Lundin *et al.*, 1969; Radford & Renard, 1984; Saccomano *et al.*, 1986) and projected substantial risks due to cumulative residential radon exposure (BIER IV 1988), evidence of effects from residential exposure is inconsistent. Several studies have suggested increased lung cancer risk related to indoor radon exposure (Axelson *et al.*, 1979; Axelson *et al.*, 1988; Edling *et al.*, 1984; Svensson *et al.*, 1987; Svensson *et al.*, 1989; Lees *et al.*, 1987; Schoenberg *et al.*, 1989). Many of these studies were small and based on indirect or short-term radiation measurements, and not all studies have demonstrated such a link (Simpson & Comstock, 1983; Damber & Larsson, 1987). Most recently, a relatively large population-based case-control study from China found no overall association between household radon exposure and lung cancer risk (Blot *et al.*, 1990).

Studies of miners do not indicate risks for other cancers, although the relatively small numbers and the overwhelming lung cancer risks make it unlikely that an effect would be seen for other tumours. In contrast, several recent ecologic studies have linked potential residential radon exposure with risk of leukaemia and other cancers (Henshaw *et al.*, 1990, Lucie 1989; Alexander *et al.*, 1990). Two of these reports suggested that effects may be more pronounced for childhood leukaemias (Alexander *et al.*, 1990; Henshaw *et al.*, 1990). Such an observation is consistent with both age-related differences in radiation sensitivity and the fact that children will have fewer residences and thus are less likely to be misclassified by a measure of radon exposure in the most recent residence.

Henshaw (1990) has suggested that the bone marrow dose from radon inhalation is sufficient to cause leukaemia, although his results have been quite controversial (e.g. Mole, 1990; Bowie, 1990). This evidence, and that from other studies potentially linking very low-dose gamma irradiation from nuclear installations and leukaemia in children (Roman *et al.*, 1987; Ewings *et al.*, 1989; Cook-Mozaffari *et al.*, 1987), offer justification for further studies of childhood cancer in relation to residential radon exposure.

We have explored the potential link between radon and childhood cancer mortality in North Carolina using county-specific levels of radon in groundwater as our indication of exposure. Levels of radon in indoor air are influenced directly by the amount of radon gas which accumulates in bedrock under a house and diffuses inside through cracks in its foundation and outer structure (Bruno, 1983). Radon may also enter a house through tap water. With agitation and heating, some fraction of the original radon in the water used in the home will diffuse into the air. It has been estimated that 10-15% of total radon in indoor air may typically be attributed directly to outgassing from tap water (Kahlos & Asikainen, 1980), and that 1-7% of all lung cancer deaths due to indoor radon are due to that same source (Cothern *et al.*, 1986).

Although waterborne radon generally makes a small contribution to the amount of radon in indoor air, houses with high radon concentrations in water have the potential for high air concentrations because both airborne and waterborne radon may have a common geological source. Furthermore, houses in areas with high concentrations of radon in groundwater have the potential for high exposure in indoor air, even if household water comes from another source. Such homes are also likely to have higher than average background levels of gamma radiation due to both the high correlation between radon levels and background gamma levels and the energy emitted during radon decay. Persons who live in areas where groundwater is not contaminated with radon will generally have no opportunity for household radon exposure. Thus, water radon concentration can serve as a marker of indoor radon exposure. Water radon should be a better marker than geology alone, which has been used in other studies (Archer, 1987; Fleischer, 1986).

### Methods

### *Radon measurements*

The concentration of radon in groundwater supplies in North Carolina was available from surveys of 308 public water supplies in communities with a population of at least 100 people. These were carried out by the North Carolina Department of Human Resources and the US Environmental Protection Agency in 1975 and 1981-2 (Sasser & Watson, 1978; Horton, 1983). The North Carolina Department of Human Resources survey tended to measure water before treatment, while the EPA study was designed to measure concentrations in water as consumed by the public. Never-

Correspondence: D.P. Sandler, Environmental and Molecular Epidemiology Section, Epidemiology Branch, MD A3-05, National Institute of Environmental Health Sciences, PO Box 12233, Research Triangle Park, NC 27709, USA.
Received: 28 March 1990; and in revised form 3 December 1990.

theless, there was no evidence of a systematic difference in the radon concentrations determined by the two surveys in 21 water supplies measured in both. Radon concentrations were approximately log-normally distributed so the geometric mean radon concentration was calculated for 75 counties where direct measurements were available for one or more water supplies. For 25 counties with missing data, radon concentration was imputed by linear regression based on radon concentrations in other North Carolina counties with similar geological characteristics (Loomis *et al.*, 1988). All counties were subsequently ranked on the basis of their geometric mean radon concentration and then divided into thirds: 0–228 pCi $l^{-1}$, 229–1375 pCi $l^{-1}$, and 1376–10,692 pCi $l^{-1}$, referred to as low, medium, and high.

Although the imputed radon values are based on geology, the modelled relationship between geology and radon levels is influenced by characteristics of soil, weather, and other features that are unique to North Carolina. Thus, the imputed values are more than an indirect measure based on rock type alone. While the observed relationships between rock type and radon concentration in North Carolina are similar to those reported elsewhere, there is enough variability to suggest that data from contiguous areas would provide a better estimate of local water radon levels than would data from more distant regions (Loomis *et al.*, 1988). Nonetheless, we repeated our analysis, restricting the data to those counties for which actual water radon measurements were available. The results were identical to those using the best available data (actual or imputed radon levels) for each of the counties in North Carolina.

*Cancer mortality rates*

Crude and age-sex-adjusted mortality rates for children under age 15 were calculated by tertile of radon exposure using 1950–1979 cancer mortality data compiled by the US EPA (Riggan *et al.*, 1983). Cancers selected were those most common in childhood: leukaemia, cancers of the brain and central nervous system, lymphosarcoma, reticulum cell sarcoma and other lymphomas, connective and soft tissue tumours, kidney cancer, and bone cancer. Rates were standardised to the 1960 US population by the direct method, using 5-year age-sex-specific groups. Relative risks compare adjusted rates in the medium and high radon concentration counties with those in the low concentration counties. Approximate 95% confidence intervals were calculated using Taylor series variance estimates (Kleinbaum *et al.*, 1982).

### Results

Adjusted childhood cancer mortality rates per 100,000 for North Carolina are given in Table I and radon-associated relative risks are shown in Table II. The relative risk for all childhood cancers combined was slightly increased for both medium (RR = 1.16, 95% confidence interval (CI) 1.05, 1.28) and high radon counties (RR = 1.23, 95% CI 1.11, 1.37).

**Table I** Age and sex adjusted mortality rates for selected childhood cancers in North Carolina 1950–1979

| *Cancer (ICD-9[a] code)* | *Deaths* | *Death rate per 100,000* |
|---|---|---|
| All cancers combined | 2706 | 17.92 |
| Leukaemias (204-8, 202.4, 203.1) | 1194 | 7.93 |
| Brain and CNS (191, 192) | 454 | 2.98 |
| Lymphomas[b] (200, 202, 159.1, 202.0, 202.1, 202.8, 202.9) | 213 | 1.40 |
| Connective and soft tissue (171, 164.1) | 176 | 1.19 |
| Kidney (189 except 189.3) | 165 | 1.12 |
| Bone (170) | 108 | 0.69 |
| All other cancers | 396 | 2.61 |

[a]ICD-9, International Classification of Diseases, Ninth Revisions. [b]Lymphosarcomas and reticulum cell sarcoma including other lymphomas.

Risk for childhood leukaemia was significantly increased in both medium and high radon counties (RR = 1.26 and RR = 1.33). Relative risks were also increased for brain and CNS tumours, lymphomas, and bone cancer, but their precision was poor and dose-response gradients were not always present.

### Discussion

Using a measure of mean radon concentration in groundwater, this study provides evidence that exposure to high levels of radon at home may increase the risk of mortality from childhood cancer. These results are consistent with those of Henshaw *et al.* (1990), Alexander *et al.* (1990), and others (Lucie, 1989; Roman *et al.*, 1987; Cook-Mozaffarri *et al.*, 1987) who have demonstrated associations between childhood cancer, particularly leukaemia, and exposure to levels of radiation previously considered to be too low to plausibly increase risk. One case-control study, on the other hand, has reported no randon exposure differences for childhood cancer cases and controls, but only 15 case-control pairs were studied (Stjernfeldt *et al.*, 1987).

As with all ecological studies, it is not possible to attribute individual deaths to radon exposure: county-specific radon concentrations, as used here, simply reflect the potential for radon exposure. In general, residences in counties with high radon concentrations in groundwater will have higher indoor radon exposures in air and/or water (Hess *et al.*, 1987). The North Carolina Agricultural Extension Service (1990) recently reported results of a survey of radon concentrations in indoor air in randomly selected houses in 16 North Carolina counties. The counties in which a high proportion

**Table II** Relative risk of dying from selected childhood cancers in relation to radon exposure

| | Radon exposure | | | | | |
|---|---|---|---|---|---|---|
| | Medium | | | High | | |
| *Cancer* | *Deaths* | *RR[a]* | *95% CI* | *Deaths* | *RR[a]* | *95% CI* |
| *North Carolina* | | | | | | |
| All cancers combined | 1301 | 1.16 | (1.05, 1.28) | 839 | 1.23 | (1.11, 1.37) |
| Leukaemias | 585 | 1.26 | (1.08, 1.47) | 375 | 1.33 | (1.13, 1.57) |
| Brain and CNS | 232 | 1.28 | (1.00, 1.62) | 130 | 1.18 | (0.90, 1.54) |
| Lymphomas[b] | 97 | 1.13 | (0.79, 1.62) | 73 | 1.38 | (0.95, 2.02) |
| Connective and soft tissue | 81 | 1.00 | (0.69, 1.46) | 54 | 1.11 | (0.74, 1.66) |
| Kidney | 74 | 0.96 | (0.65, 1.41) | 52 | 1.13 | (0.74, 1.70) |
| Bone | 55 | 1.22 | (0.75, 1.98) | 30 | 1.06 | (0.62, 1.83) |
| Other cancers | 177 | 0.95 | (0.74, 1.21) | 125 | 1.10 | (0.85, 1.44) |

[a]Relative risk comparing potentially exposed (medium or high) counties to counties with low radon levels. [b]Lymphosarcomas and reticulum cell sarcomas including other lymphomas.

of homes were found to have high radon levels were counties we identified as having high concentrations of radon in groundwater.

At least half of the people in North Carolina are served by public water supplies (NC Department of Water Resources 1961). Of these, only about 15% are from groundwater. Including the population not served by public supplies, however, 59% of the state uses groundwater. Groundwater public supplies predominate in the eastern (Coastal Plain) portion of the state. Elsewhere, public supplies use mostly surface water, yet houses not served by public supplies in these areas also use groundwater.

The degree of correlation between radon levels in public groundwater supplies and in private wells in the same area and the proportion of homes within each county served by groundwater were not taken into account in our analysis. Any resulting misclassification should, however, have reduced our ability to detect differences. Our results reflect qualitative rather than quantitative relationships between waterborne radon and childhood cancer. The data suggest increased cancer risk associated with increasing levels of radon in groundwater. However, because of uncertainties in the measure of radon exposure, we are unable to quantify the actual levels at which effects are seen. We have used groundwater radon measurements simply as a tool for roughly characterising the potential for radon exposure. Since measurements were available only for radon in water, our data underestimate total radon exposure by ignoring the potentially much larger contributions from other sources.

Water radon concentrations were determined in 1975 and later whereas cancer mortality data spanned the period 1950 to 1979. Although it is not likely that there have been major geologic changes which would have caused radon levels to change over time, tap water sources and residential areas may have changed during this period. A number of areas previously served by groundwater may now be served by surface water or different groundwater supplies. Change is most likely to have occurred in previously rural areas or suburban communities incorporated into neighbouring metropolitan areas. To the extent that we are estimating risk from drinking water *per se*, this increases the likelihood of misclassification that would result in bias towards the null. Similarly, the reliance on a one time measure of potential radon exposure (in the residence at time of death for cancer cases) also introduces potential misclassification.

The counties with high radon concentration in groundwater tend to be located in the western and central portions of the state (Collman *et al.*, 1988). It is possible that there is some unknown confounding factor that also has this geographic distribution. We know of no common industrial or other exposure in these counties, however, that could explain the childhood cancer findings.

During the time period of study, dramatic improvements took place in the treatment of childhood leukaemia. If areas with higher radon levels were also those least likely to have access to medical improvements, a spurious association between radon levels and mortality would occur. However, three out of four major medical centres in North Carolina are located in counties with medium or high groundwater radon concentrations. Furthermore, treatment advances were not really apparent until the mid to late 1970s. Any potential biases due to differential medical care or survival would have only a minimal effect on our data, the bulk of which was from an earlier time period.

The association between radon as measured in groundwater and cancer risk for children, while open to alternative explanations, is, together with the results of other surveys, cause for concern about the potentially broad consequences of exposure to low-level radiation in early life. We join others (Peto, 1990) in urging that this be tested in a more rigorous study before being dismissed, a priori, as implausible.

### References

ALEXANDER, F.E., MCKINNEY, P.A. & CARTWRIGHT, R.A. (1990). Radon and leukaemia. *Lancet*, **i**: 1336.

ARCHER, V.E. (1987). Association of lung cancer mortality with precambrian granite. *Arch. Environ. Health*, **42**, 87.

AXELSON, O., EDLING, C. & KLING, H. (1979). Lung cancer and residency – a case-referent study on the possible impact of exposure to radon and its daughters in dwellings. *Scand. J. Work Environ. Health*, **5**, 10.

AXELSON, O., ANDERSSON, K., DESAI, G. & 4 others (1988). Indoor Radon exposure and active and passive smoking in relation to the occurrence of lung cancer. *Scand. J. Work Environ. Health*, **14**, 286.

BLOT, W.J., XU, Z.Y., BOICE, J.D. & 5 others (1990). Indoor radon and lung cancer in China. *J. Natl Cancer Inst.*, **82**, 1025.

BOWIE, S.H.U. (1990). Radon and leukemia. *Lancet*, **i**, 1336.

BRUNO, R.C. (1983). Sources of indoor radon in houses: A review. *J. Air Poll. Control Assoc.*, **33**, 105.

COLLMAN, G.W., LOOMIS, D.P. & SANDLER, D.P. (1988). Radon-222 concentration in groundwater and cancer mortality in North Carolina. *Int. Arch. Occup. Environ. Health*, **61**, 13.

COMMITTEE ON THE BIOLOGIC EFFECTS OF IONIZING RADIATION (1988). *BIER IV. Health risks of radon and other internally deposited alpha-emitters*. National Academy Press: Washington, DC.

COOK-MOZAFFARI, R.J., ASHWOOD, F.L., VINCENT, T., FORMAN, D. & ALDERSON, M. (1987). *Cancer incidence and mortality in the vicinity of nuclear installations, England and Wales 1959–80* (Stud. Med. Popul. Subj. no. 51). HM Stationery Office: London.

COTHERN, C.R., LAPPENBUSCH, W.L. & MICHEL, J. (1986). Drinking-water contribution to natural background radiation. *Health Phys.*, **50**, 33.

DAMBER, L.A. & LARSSON, L.G. (1987). Lung cancer in males and type of dwelling. An epidemiologic pilot study. *Acta Oncol.*, **26**, 25.

EDLING, C., KLING, H. & AXELSON, O. (1984). Radon in homes – A possible cause of lung cancer. *Scand. J. Work Environ. Health*, **10**, 25.

EWINGS, P.D., BOWIE, C., PHILLIPS, M.J. & JOHNSON, S.A.N. (1989). Incidence of leukaemia in young people in the vicinity of Hinkley Point nuclear power station, 1959–86. *Brit. Med. J.*, **299**, 289.

FLEISCHER, R.L. (1986). A possible association between lung cancer and a geological outcrop. *Health Phys.*, **50**, 823.

HENSHAW, D.L., EATOUGH, J.P. & RICHARDSON, R.B. (1990). Radon as a causative factor in induction of myeloid leukemia and other cancers. *Lancet*, **i**, 1008.

HESS, C.T., KORSAH, J.K. & EINLOTH, C.J. (1987). Radon in houses due to radon in potable water. In *Radon and its Decay Products. Occurrence, Properties, and Health Effects*, Hopke, P.K. (ed.) p. 30. ACS Symposium Series 331. American Chemical Society: Washington, DC.

HORTON, T.R. (1983). *Methods and results of EPA's study of radon in drinking water*. US Environmental Protection Agency, EPA 520/5-83-027.

KAHLOS, H. & ASIKAINEN, M. (1980). Internal radiation doses from radioactivity of drinking water in Finland. *Health Phys.*, **39**, 108.

KLEINBAUM, G., KUPPER, L. & MORGENSTERN, H. (1982). *Epidemiologic Research. Principles and Quantitative Methods*. Lifetime Learning Press, Wadsworth, Inc.: California.

LEES, R.E., STEELE, R. & ROBERTS, J.H. (1987). A case-control study of lung cancer relative to domestic radon exposure. *Int. J. Epidemiol.*, **16**, 7.

LOOMIS, D.P., WATSON, J.E. & CRAWFORD-BROWN, D.J. (1988). Predicting the occurrence of radon-222 in groundwater supplies. *Environ. Geochem & Health*, **10**, 41.

LUCIE, N.P. (1989). Radon exposure and leukaemia. *Lancet*, **ii**, 99.

LUNDIN, F.E., LLOYD, J.W., SMITH, E.M., ARCHER, V.E. & HOLADAY, D.A. (1969). Mortality of uranium miners in relation to radiation exposure, hard rock mining and cigarette smoking-1950 through September 1967. *Health Phys.*, **16**, 571.

MOLE, M.H. (1990). Radon and leukemia. *Lancet*, **i**, 1336.

NORTH CAROLINA AGRICULTURAL EXTENSION SERVICE, HOME ECONOMICS DEPARTMENT (1990). *North Carolina Agricultural Extension Service Radon Study*. Raleigh, NC (R.W. Leker, personal communication).

NORTH CAROLINA DEPARTMENT OF WATER RESOURCES, DIVISION OF STREAM SANITATION AND HYDROLOGY (1961). *Bulletin 2. Chemical and Physical Character of Municipal Water Supplies in North Carolina*. NC Department of Water Resources: Raleigh.

PETO, J. (1990). Radon and the risks of cancer. *Nature*, **345**, 389.

RADFORD, E.P. & RENARD, K.G. (1984). Lung cancer in Swedish iron miners exposed to low doses of radon daughters. *N. Engl J. Med.*, **310**, 1485.

RIGGAN, W.B., VAN BRUGGEN, J., ACQUAVELLA, J.F., BEAUBIER, J. & MASON, T.J. (1983). *US Cancer mortality rates and trends, 1950–1979*. Vol I, II, III, EPA-600/1-83-015a.

ROMAN, E., BERAL, V., CARPENTER, L. & 4 others (1987). Childhood leukaemia in the West Berkshire and Basingstoke and North Hampshire district health authorities in relation to nuclear establishments in the vicinity. *Brit. Med. J.*, **294**, 597.

RONCA-BATTISTA, M., MOON, M., BERGSTEN, J., WHITE, S.B., ALEXANDER, B. & HOLT, N. (1988). Radon-222 concentrations in the United States – Results of sample surveys in five states. *Radiation Prot. Dosim.*, **24**, 307.

SACCOMANO, G., YALE, C., DIXON, W., AUERBACH, O. & HUTH, G.C. (1986). An epidemiological analysis of the relationship between exposure to Rn progeny, smoking and bronchogenic carcinoma in the U-mining population of the Colorado plateau – 1960–1980. *Health Phys.*, **50**, 605.

SASSER, M.K. & WATSON, J.E. (1978). An evaluation of the radon concentration of North Carolina ground water. *Health Phys.*, **34**, 667.

SCHOENBERG, J.B., KLOTZ, J.B. & WILCOX, H.B. (1989). Lung cancer and exposure to radon in women in New Jersey. *MMWR*, **42**, 715.

SIMPSON, S.G. & COMSTOCK, G.W. (1983). Lung cancer and housing characteristics. *Arch. Environ. Health*, **38**, 248.

STJERNFELDT, M., SAMUELSSON, L. & LUDVIGSSON, J. (1987). Radiation in dwellings and cancer in children. *Pediatric Hematol. Oncol.*, **4**, 55.

SVENSSON, C., EKLUND, G. & PERSHAGEN, G. (1987). Indoor exposure to radon from the ground and bronchial cancer in women. *Int. Arch. Occup. Environ. Health*, **59**, 123.

SVENSSON, C., PERSHAGEN, G. & KLOMINEK, J. (1989). Lung cancer in women and type of dwelling in relation to radon exposure. *Cancer Res.*, **49**, 1861.